# Hypoglycemic efficacy of *Rosmarinus officinalis* and/or *Ocimum basilicum* leaves powder as a promising clinico-nutritional management tool for diabetes mellitus in Rottweiler dogs

**DOI:** 10.14202/vetworld.2020.73-79

**Published:** 2020-01-10

**Authors:** Noha Abdelrahman, Ramadan El-Banna, Mahmoud M. Arafa, Maha M. Hady

**Affiliations:** 1 Department of Chemistry and Toxicology, Malnutrition Unit, Animal Health Research Institute, Giza, Egypt; 2 Department of Nutrition and Clinical Nutrition, Faculty of Veterinary Medicine, Cairo University, Giza, Egypt

**Keywords:** amylase, basil, cortisol, hypoglycemia, insulin, rosemary, Rottweiler

## Abstract

**Background and Aim:**

This study examined the impact of dietary fortification with rosemary (*Rosmarinus officinalis*) and/or basil (*Ocimum basilicum*) leaves powder on glycemic status of dogs.

**Materials and Methods:**

Forty-five Rottweiler dogs were assigned to five experimental groups and fed an experimentally processed extruded basal diet that was either fortified or not fortified. G1 was fed the basal diet without any fortification (negative control); G2 was consumed the basal diet supplemented with a commercially available synthetic palatant (positive control); G3 was provided with rosemary fortified (at 0.05%) basal diet; G4 was provided with a basil fortified (at 0.05%) basal diet; and G5 was offered a rosemary and basil fortified (each at 0.025%) basal diet.

**Results:**

G4 and G5 exhibited a positive impact on growth performance traits. Dogs in G3, G4, and G5 showed significant decreases in serum glucose levels in comparison to dogs of the control groups (G1 and G2). It was clear that the inclusion level of 0.05% of basil leaves powder showed the greatest hypoglycemic action. Indeed, G4 dogs showed a reduction in blood glucose at a percentage of approximately 31% followed by G5 and G3 groups (16.25% and 14%, respectively). Furthermore, basil leaves inhibited the amylase enzyme activity. Both insulin and cortisol levels in G4 dogs were increased and reduced compared to controls, respectively. In addition, dietary fortification with rosemary and/or basil significantly increased glutathione, superoxide dismutase, and catalase levels, while values for malondialdehyde and lactate dehydrogenase were decreased.

**Conclusion:**

It could be concluded that dietary fortification of dog diet with rosemary and/or basil leaves powder at 0.05% separately or 0.025% in combination might be used as promising modulators of blood glucose levels as well as clinico-nutritional management tools for the prevention and control of diabetes mellitus in dogs.

## Introduction

Diabetes mellitus (DM) is a serious metabolic disease that is rising in prevalence worldwide in both humans and pets. It is characterized by persistent hyperglycemia and is caused either by inadequate insulin secretion from pancreatic beta cells, impaired insulin signaling, or both. DM is one of the most frequently diagnosed endocrine disorders in dogs and cats. More than 95% of diabetic dogs are affected by Type I DM versus 20% of diabetic cats [1,2]. Despite considerable progress in the treatment of diabetes with synthetic drugs, their associated adverse effects and sometimes reduced effectiveness overtime remain a major challenge. Thus, there is an ongoing effort to identify natural, effective, and inexpensive agents with antihyperglycemic and antidyslipidemic properties and minimal side effects.

Medicinal herbs have been used to prevent and/or control metabolic, nutritional, and health-related diseases in both humans and animals. They possess pharmacological impacts as anti-inflammatory, antioxidant, antimicrobial, antispasmodic, anti-allergic, anticancer, antiaging, hypolipidemic, hepatoprotective, neuroprotective, hypotensive, hypoglycemic, central nervous system– stimulant, and analgesic agents [3]. Treatment with synthetic pharmaceutical compounds is usually associated with side effects, but natural, medicinal plants are regarded as promising therapeutic agents with more minimal side effects. In addition to the more favorable safety profile, they are less expensive than synthetic medications which lessen the cost of health care [4]. According to Viegi *et al*. [5] who carried out a veterinary ethnobotanical study in Italy, dogs represent 5.3% of all domestic animals treated with medicinal plants. Although the utilization of phytotherapeutic agents is rising in companion animals, there are few clinical studies related to the therapeutic use of these remedies in pets [3].

Interestingly, rosemary and basil possess antihyperglycemic and antidyslipidemic effects and they could be used as promising natural remedies that can prevent and control DM in humans and pets [6,7]. Rosemary contains many biologically active compounds, including volatile fractions and a variety of phenolic compounds that exert multiple biological activities, including hypoglycemic, antioxidant, cholagogue, choleretic, antidiuretic, anti-inflammatory, hypolipidemic, and hepatotonic properties [7,8]. In addition, Labban *et. al*. [9] offered three different doses of dried rosemary leaves (2, 5 and 10 g/d) to three groups of people. They noticed a significant decrease in blood glucose level in a dose-dependent manner (11.2%, 15.74% and 18.25% for 2, 5 and 10 g/d, respectively). A previous study [10] evaluated the influence of aqueous rosemary extract (RE) on streptozotocin-induced diabetic rats. Rats were provided with a commercial diet supplemented with 200 mg/kg body weight (BW) of RE for 3 weeks. The results implied that RE significantly decreased blood glucose levels by 36.9% compared to the control condition. Other authors carried out a study using streptozotocin-induced Type 1 diabetic rats and high-fat diet-induced Type 2 diabetic rats to investigate the impact of rosmarinic acid (RA) on glucose homeostasis. They used different doses of RA (120, 160, and 200 mg/kg BW) for 28 days. Their results revealed that RA reduced postprandial glucose level in a dose-dependent manner [11]. In addition to rosemary, basil (*Ocimum basilicum*) has long been used in traditional herbal medicine for the management of diabetes [12]. Recently, Ezeani *et al*. [13] supplemented groups of diabetic rats with basil extract at different concentrations (100, 200, and 400 mg/kg BW) for 2 weeks and observed a dose-dependent hypoglycemic effect. Furthermore, another study [14] showed a significant reduction in both fasting and postprandial blood glucose levels (21.75% and 28.29%, respectively) in Type 2 diabetic patients who received 30 days of basil supplementation in capsule form. Notably, the potency of these effects increased over time, as the reductions in fasting and postprandial blood glucose levels (39.66% and 43.68%) were greater after 90 days of supplementation versus that after 30 days. Moreover, the glycosylated hemoglobin reduction percentage was reported to be significantly reduced (35.82%) by the end of the study.

A study was conducted to evaluate the influence of basil (*Ocimum sanctum*) aqueous extract on blood glucose level using diabetic rats. Three groups of rats were used(normal control, diabetic induced non supplemented and diabetic-induced rats supplemented with 5.9 g/kg BW of *O. sanctu*m aqueous extract for 3 weeks). As indicated, basil extract significantly reduced blood glucose level and a possible explanation is its positive effects on pancreatic β-cells function. Their assumption was supported by the increased insulin level in diabetic rats treated with the extract [15].

Despite the known hypoglycemic effects of rosemary and basil, there are few studies that have examined the impact of these and other phytogenic food additives on DM in pets. Therefore, this study was carried out to explore the hypoglycemic effects of both *Rosmarinus officinalis* and *O. basilicum* leaves powder in Rottweiler dogs and to recommend specific levels of supplementation for each herb in dog diets for potential use as natural, phytogenic, and palatable food additives to reduce glucose levels.

## Materials and Methods

### Ethical approval

The study was approved by the Institutional Animal Care and Use Committee (IACUC), Cairo University, Egypt with Protocol number CU II F 18 18.

### Study area

The current study was carried out at a private dog farm located in Cairo, Egypt, from November 2017 to January 2018. The study was conducted to examine the impact of rosemary (*R. officinalis*) and/or basil (*O. basilicum*) leaves powder (added to Rottweilers’ food at a rate of 0.025-0.05%) on growth performance parameters (weight gain, actual food intake, and refusal), selected serum parameters including glucose, insulin, cortisol, and amylase (a pancreatic enzyme), and antioxidant biomarkers (glutathione [GSH], catalase [CAT], superoxide dismutase [SOD], lactate dehydrogenase [LDH], and malondialdehyde [MDA]) in Rottweiler dogs.

### Preparation of rosemary and basil leaves powder

Rosemary and basil leaves were collected in September 2017 from medicinal plants farm located in Egypt. The leaves were washed of residual soil, completely dried, ground to fine powder, and stored in tightly closed plastic containers until use.

### Animals, housing, and management

A total of 45 Rottweiler dogs (BW within 20.5 and 24.5 kg) were utilized throughout this study. The dogs were assigned to five experimental groups with three replicates (three dogs) each; each group included approximately equal numbers of males and females (nine dogs divided into five males and four females). Moreover, the age of dogs was about 4 months at the beginning of the study which lasted for 8 weeks (dogs reached 6 months of age). As known, sex hormones still of in-noticeable levels in blood until the age of maturity (12 months). In other words, there was no impact of sex in this study because dogs were in puppy stage not in adult one.

All dogs were vaccinated with Vanguard^®^ vaccine and dewormed using Drontal Plus^®^ before the experiment. Animals were housed in individual kennels (1 m×1.2 m) and had access to an outside kennel (10 m×20 m) for exercise and socialization with each other for 1 h daily. Freshwater was available *ad libitum* throughout the experiment. Kennels were cleaned twice daily. All animals were showered once weekly with Betadine^®^ shampoo and Cytéal^®^ antiseptic foaming solution. The experimental feeding study lasted for 8 weeks in addition to a 2-week preliminary period for acclimatization.

### Experimental diets

An isonitrogenous equicaloric basal diet was formulated on the basis of the actual proximate chemical composition (AOAC) [16] of the locally available raw materials used in the diet formula. All dietary ingredients used were locally prepared and processed in an extruded form (with a single-screw extruder at Al-Okhwa factory, Kafr El-Sheikh Governorate, Egypt), and rosemary and/or basil leaves powder was supplemented at different proportions during the coating step of diet manufacture. All analytical procedures of dietary ingredients, food additives, and final processed extruded diets were carried out at the Regional Center for Food and Feed; Agricultural Research Center, Giza, Egypt. Each dog in the different experimental groups was fed separately (based on the energy distribution recommendation of the Association of American Feed Control Officials [17], and the amount of food provided daily was calculated according to the dog’s BW, energy requirements, and the energy density of the diet using the following equations [18]:

Resting energy requirement (RER)=(30×BW)+70 (kcal).

Metabolizable energy requirement (MER)= RER×2 (kcal).

Daily energy requirement=MER×1.5 (kcal).

The energy density of the food was calculated through the following equation (2):

ME of food=(CP%×3.5)+(NFE%×3.5)+ (EE%×8.5) kcal/100 g food

Isocaloric expression indicates that each dog was fed according to its energy requirements depending on its BW, but it does not refer to the same energy density of the diets. The amount of daily food for each dog in the five experimental groups was weighed and divided into two equal portions and fed at 9:00 AM and 5:00 PM in a stainless steel bowl. Each dog was allowed 30 min to consume the food; then, the bowls were removed, and any residual food from the previous meal was collected and weighed before the next meal. The ingredients in each of the five experimental diets are summarized in Table-1. The results of the nutrient contents of the food additives and dietary ingredients and the chemical analysis of the experimental basal diet are presented in Tables-2 and 3 [2].

**Table-1:** Ingredient composition of the experimental diets.

Ingredient (%)	G1	G2	G3	G4	G5
Chicken meal	25	25	25	25	25
Soybean meal	17	17	17	17	17
Corn gluten	8	8	8	8	8
Yellow corn	32.6	32.6	32.6	32.6	32.6
Beet pulp	5	5	5	5	5
Soybean oil	7	7	7	7	7
Linseed oil	3	3	3	3	3
**Syn. Pal.	-	0.05			
*ROS.L.P	-	-	0.05	-	0.025
*BAS.L.P	-	-	-	0.05	0.025
NaCl	0.3	0.3	0.3	0.3	0.3
CaCO_3_	0.5	0.5	0.5	0.5	0.5
MCP	1	1	1	1	1
Premix	0.4	0.4	0.4	0.4	0.4
Mold inhibitor	0.1	0.1	0.1	0.1	0.1
Toxin binder	0.1	0.1	0.1	0.1	0.1

* ROS.L.P=Rosemary leaves powder, BAS.L.P=Basil leaves powder,

** Syn. Pal=Synthetic palatant, G1= Group 1, G2=Group 2, G3=Group 3, G4=Group 4, G5=Group 5, NaCl=Sodium chloride, CaCO_3_=Calcium carbonate, MCP=Monocalcium phosphate. Premix=Each 4 kg contains the following: Vitamin A 19,000,000 IU, Vitamin D3 1,900,000 IU, Vitamin E 100,000 mg, Vitamin K3 4000 mg, Vitamin B1 5000 mg, Vitamin B2 7000 mg, Vitamin B6 5000 mg, Vitamin B12 40 mg, niacin 35,000 mg, biotin 250 mg, folic acid 2000 mg, pantothenic acid 25,000 mg, zinc 120,000 mg, Mn 40,000 mg, iron 200,000 mg, copper 20,000 mg, iodine 2000 mg, cobalt 4000 mg, selenium 350 mg, BHT 180,000 mg, and CaCO_3_ up to 4 kg

**Table-2:** Chemical composition of the basal diet used in the experiment.

DM%	CP%	EE %	CF%	Ash%	NFE%	*ME (kcal/100 g)
92.5±0.5	25.3±0.44	16.23±0.28	3.2±0.26	6.54±0.1	41.23±0.24	370.81

DM=Dry matter, CP=Crude protein, EE=Ether extract, CF=Crude fiber, NFE=Nitrogen-free extract, ME=Metabolizable energy.

* Calculated according to Case *et al*. [2]

**Table-3:** Proximate analysis of additive leaves used in the experiment.

Nutrient %	Rosemary leaves	Basil leaves
Moisture	90.86±0.41	90.11±0.27
Crude protein	4±0.14	14±0.34
Ether extract	4.92±0.20	0.58±0.21
Crude fiber	16.51±0.11	7.34±0.18
Ash	6.15±0.23	12.44±0.19
Nitrogen-free extract	59.28±0.6	55.75±0.23

### Experimental design

The experimental groups were as follows: The first group (G1) was fed the basal diet without any fortification and served as the control group; the second group (G2) was fed the basal diet supplemented with a synthetic palatable flavor (PALASURANCE^®^; KEMIN Co.) at a rate of 1% and served as the positive control group. The third group (G3) was fed the basal diet that was fortified on the surface (during the coating step) with rosemary leaves powder at 0.05%. The fourth group (G4) was fed the basal diet that was fortified on the surface with basil leaves powder (0.05%). Furthermore, the fifth group (G5) was fed the basal diet fortified on the surface with both rosemary and basil leaves powder at 0.025% each.

### Growth performance parameters

Dogs in different groups were weighed individually after fasting overnight at the onset of the feeding trial and then weighed on a biweekly basis throughout the feeding trial. In addition to weight gain, each animal’s actual daily food consumption was recorded, as well as the amount of food remnants after each meal.

### Serum parameters and antioxidant biomarkers

After dogs were fasted overnight (to avoid any dietetic effect on serum components), blood samples were collected through cephalic vein puncture from two dogs in each replicate, and sera were separated, refrigerated at −20°C, and subsequently analyzed for serum glucose, insulin, cortisol, amylase, reduced GSH, SOD, CAT, LDH, and lipid peroxidation “MDA” levels using commercial kits (Biodiagnostic) at the Al-Nile Laboratory, Egypt.

### Statistical analysis

Statistical analyses were performed with SPSS 20 software (IBM, NY, USA). The results are expressed as treatment means with their pooled standard error of means. The data were analyzed by one-way analysis of variance and p>0.05 was considered as statistically significant.

## Results

### Growth performance parameters of dogs

The results of the impact of diet fortification with dried rosemary and/or basil leaves powder on growth performance parameters of Rottweiler dogs are presented in Table-4. Dogs of groups G2 (basal diet plus synthetic flavor) and G5 (basal diet plus 0.025% each rosemary and basil) gained the most weight and showed the highest actual food intake (and thus lowest amount of food remnants) in comparison to the other groups followed by dogs in G4 (basal diet plus 0.05% basil) and G1 (basal diet control). However, dogs in G3 (basal diet plus 0.05% rosemary) showed pronounced weight loss and the highest rates of food refusal among all groups at the end of the experimental period.

**Table-4:** Growth performance parameters (mean±standard error) in different experimental groups at the end of the 8-week experiment.

Parameter	G1	G2	G3	G4	G5	p-value
Initial weight (kg)	20.16±0.320	24.24±0.228	21.02±0.401	23.17±0.250	23.37±0.210	-
Final weight (kg)	23.35±0.12	27.83±0.1	21.6±0.2	24.86±0.15	28.44±0.13	-
Weight change (kg)	2.85^c^±0.02	5.83^a^±0.02	−2.9^d^±0.01	3.86^b^±0.03	5.44^a^±0.1	<0.001
Actual intake (kg)	29.47^cd^±0.36	37.96^a^±0.23	26.59^d^±1.85	33.33^bc^±0.77	36.18^a^b±0.3	<0.001
Refusal amount (kg)	3.29^b^±0.25	0.74^c^±0.03	7.43^a^±0.54	3.86^b^±0.22	1.26^c^±0.09	<0.001

a,b,c,d Means with different letters within the same row are significantly different at p≤0.05. G1=Group 1, G2=Group 2, G3=Group 3, G4=Group 4, G5=Group 5

### Selected serum parameters of dogs

The results illustrating the impact of dried rosemary and/or basil leaves powder on serum glucose levels are presented in Table-5. We found that dietary fortification with rosemary leaves (G3) or basil leaves (G4) separately at 0.05% or in combination at 0.025% each (G5) significantly reduced circulating glucose levels (p≤0.05) in comparison to the controls (G1 and G2). It was clear that dogs fed with the dried basil leaves powder supplemented diet at the inclusion level of 0.05% showed the greatest hypoglycemic action by a reduction of approximately 31% followed by the counterpart mixture of rosemary and basil and rosemary alone (16.25% and 14%, respectively).

**Table-5:** Levels of selected serum parameters (mean±standard error) in different experimental groups at the end of the 8-week experiment.

Parameter	G1	G2	G3	G4	G5	p-value
Glucose (mg/dl)	116.97^a^±1.11	117.7^a^±1.14	100.53^b^±2.62	80.68^c^±5.62	97.96^b^±2.14	<0.001
Insulin (IU/ml)	3^c^±0.06	3.1^c^±0.05	3.57^c^±0.16	5.36^b^±0.25	6.07^a^±0.13	<0.001
Cortisol (µg/dl)	12.06^a^±0.22	12.15^a^±0.2	10.79^b^±0.41	10.49^b^±0.41	8.25^c^±0.11	<0.001
Amylase (U/L)	652.22^a^±1.2	653.44^a^±1.13	649.78^a^±14.61	571.56^b^±12.85	635.22^a^±4.22	<0.001
Glutathione (U/ml)	12.6^c^±0.55	12.8^c^±0.55	15.67^b^±0.21	15.65^b^±0.22	18.26^a^±0.21	<0.001
Catalase (U/ml)	22.17^d^±0.41	23.03^d^±0.43	39.49^a^±0.36	28.73^b^±0.51	26.72^c^±0.62	<0.001
Superoxide dismutase (U/ml)	23.67^b^±0.67	24.06^b^±0.7	28.84^a^±0.64	29^a^±0.41	30.57^a^±0.28	<0.001
Lactate dehydrogenase (U/ml)	57.94^a^±0.69	58.56^a^±0.73	33.94^b^±1.29	39.44^b^±2.37	36.11^b^±0.95	<0.001
Malondialdehyde (U/ml)	13.76^a^±0.26	14.02^a^±0.28	10.98^b^±0.23	10.41^b^±0.23	7.6^c^±0.09	<0.001

a,b,c,d Means with different letters within the same row are significantly different at p≤0.05. G1=Group 1, G2=Group 2, G3=Group 3, G4=Group 4, G5=Group 5

## Discussion

### Growth performance parameters of dogs

As indicated in Table-4, dogs in Groups G2 (basal diet plus synthetic, palatable flavor) and G5 (basal diet plus 0.025% each rosemary and basil) gained more weight and showed greater actual food intake (and thus lower amount of food remnants) in comparison to the other groups followed by dogs in G4 (basal diet plus 0.05% basil) and G1 (basal diet control). However, dogs in G3 (basal diet plus 0.05% rosemary) showed pronounced weight loss and the highest rates of food refusal among all groups at the end of the experimental period. The higher final BW and food intake observed in G2 and G5 animals could be attributed, in part, to the effect of the synthetic food palatant (G2) and the possible palatant effects of equal amounts of rosemary and basil leaves powder at 0.025% each (G5). However, higher levels of individual rosemary and basil fortification resulted in reduced food intake and food palatability, and acceptability, consequently, affected the final weight gain. Palatability is one of the most important issues in pet food production as it is positively correlated with food consumption and thus animal health and performance, especially in dogs that are picky eaters. Pet food producers depend on the use of suitable and attractive flavors and/or digest as selling points for their products. Palatability is regarded as a critical concern in pet food, as it affects food attractiveness, food intake, and refusal; consequently, it affects animal health and performance. Unfortunately, the Ministry of Agriculture and Organization of Export and Import in Egypt applied restrictions regarding the importation of flavor, a situation that made the production of acceptable and palatable pet food more difficult. These restrictions necessitate the identification of natural flavoring agents to overcome such food palatability problems. We hope that the sole use of basil or its combination with rosemary leaves powder in the current study may play an important role as an attractive palatant for dog food in addition to its clinico-nutritional role in some physiological, nutritional, and health-related problems as hyperglycemia, hyperlipidemia, and obesity.

### Selected serum parameters of dogs

The results illustrating the impact of dried rosemary and/or basil leaves powder on serum glucose levels are presented in Table-5. We found that dietary fortification with rosemary leaves (G3) or basil leaves (G4) separately at 0.05% or in combination at 0.025% each (G5) significantly reduced circulating glucose levels (p≤0.05) in comparison to the control groups (G1 and G2). It was clear that the inclusion level of 0.05% dried basil leaves powder showed the greatest hypoglycemic action by a reduction of approximately 31% followed by the counterpart mixture of rosemary and basil and rosemary alone (16.25% and 14%, respectively). The increased hypoglycemic impact observed in G4 compared to G3 and G5 may be related to either the type or concentration of phenolic compounds found in basil. These compounds may be more potent in reducing serum glucose levels than those found in rosemary.

The hypoglycemic effects of rosemary and/or basil might be due to their positive impact on pancreatic β-cells function, either through direct or indirect actions [19,20]. The direct actions might be triggered through a stimulatory effect of the rosemary and basil on insulin secretion, as presented in Table-5. Indeed, the elevation of serum insulin levels stimulates glucose utilization by peripheral tissues and glucose uptake by cells, consequently leading to a reduction in serum glucose levels. In addition, the reduction in glucose levels resulting from dietary supplementary with basil leaves alone could be possibly attributed to the inhibition of the carbohydrate metabolic pathway through amylase enzyme reduction(Table-5). Accordingly, the inhibition of carbohydrates breakdown results in impeded glucose release and, consequently, a reduction in serum glucose levels [20,21]. It is worth mentioning that the suggested amylase reduction pattern is more likely to be a mode of action for hypoglycemia in the group fed the basil leaves supplemented diet (G4). Amylase levels in both G3 and G5 were marginally lower than those in the control groups, without a significant difference compared to basil leaves – supplemented group (G4).

In addition, basil and rosemary might potentiate insulin secretion indirectly through their antioxidant properties. They contain polyphenolic compounds and flavonoids which act as free radical scavengers [19]. In turn, these compounds prevent tissue degeneration and, consequently, prevent progressive β-cell impairment; hence, glycemic status is improved [20]. The speculated antioxidant hypoglycemic hypothesis was validated by oxidative stress biomarker results (Table-5). GSH, SOD, and CAT were significantly increased in all experimental groups. Moreover, MDA and LDH decreased. It is worth mentioning that the suggested hyperinsulinemic pathway is more likely to be involved in both G4 and G5 groups than G3 group. Insulin levels in rosemary-supplemented dogs were marginally higher than those in the control dogs and were not significantly different compared to dogs in G4 and G5. In addition, reduced cortisol levels (Table-5) might be the second possible mechanism of action by which hypoglycemia was induced. Rosemary and/or basil supplementation significantly (p≤0.05) decreased cortisol levels in all experimental groups. It is a well-established fact that cortisol is negatively associated with both insulin secretion and sensitivity, while it is positively correlated with insulin resistance [22]. Therefore, glucose levels might be reduced indirectly through the adrenal corticoid-glycemic pathway either by increased insulin secretion (Table-5) or enhanced insulin sensitivity.

Our findings are in accordance with those of Labban *et al*. [9] and Hannan *et al*. [21] who recorded the hypoglycemic influence of rosemary and basil leaves powder in rats and humans, respectively. Moreover, others reported that RE potently reduced serum glucose in rats [10,19]. Furthermore, our data are supported by several previous studies that reported the ability of basil extract to decrease blood glucose levels [13,21].

The previous study speculated similar mechanisms of action by which rosemary and/or basil exhibited a hypoglycemic influence. Rosemary leaves powder reduced serum glucose levels in humans through its stimulatory action on exogenous insulin secretion [9]. Moreover, the same mechanism of action was reported by other researchers that examined the hypoglycemic impact of basil extract [13,21] in their studies of the hypoglycemic impact of basil extract.

Our hypothesis concerning an antioxidant-hypoglycemic mechanism was also confirmed by the previous study. Emam [19] attributed the hypoglycemic influence of aqueous RE on rats to increased total antioxidant capacity. Accordingly, inhibition of tissue damage and potentiation of pancreatic β-cell secretion were observed. The antioxidant potency of the polyphenolic compounds and flavonoids found in basil prevented the progressive impairment of pancreatic β-cell function in their *in vitro* study [20].

The ability of basil to reduce the rates of carbohydrate metabolism and glucose release through amylase inhibitory activity has also previously been illustrated [20,21]. Moreover, a study [13] supported our hypothesis on the hypoglycemic effect induced by basil through inhibition of cortisol activity in mice. Indeed, the authors stated that basil could ameliorate adrenal corticoid-induced hyperglycemia.

## Conclusion

Our results suggest that dietary fortification of dog food with *R. officinalis* and/or *O. basilicum* leaves powder at 0.05% separately or at 0.025% each in combination might be used as a promising clinico-nutritional management tool for the prevention and control of DM in Rottweiler dogs. Consequently, specific food formulae could be suggested for practical usage in dog food. Indeed, we found that rosemary and basil not only have an impact (either negative for rosemary or positive for basil) on dog growth performance parameters but also can modulate blood glucose levels and have a positive impact on antioxidant status, as indicated by increased levels of antioxidant biomarkers.

## Authors’ Contributions

NA suggested the idea of the study, formulated different diets of the study, performed blood sampling and body weight records for dogs, supervised the processing of different extruded diets, and prepared diet portions on weekly basis based on body weight change. RE designed the proposal of the study and participated in the paper final revision and writing. MMA participated in designing of proposal and analyzed all blood and serum parameters at Al-Nile Laboratory. MMH participated in designing of proposal. All authors read and approved the final manuscript.

## References

[1] Rand J.S, Fleeman L.M, Farrow H.A (2004). Canine and feline diabetes mellitus:Nature or nurture?. J Nutr.

[2] Case L.P, Hayek M.G, Daristotle L (2011). Energy and water. Hand Book of Canine and Feline Nutrition.

[3] Tilford G.L, Wulff M.L (2009). Herbs for Pets Hand Book.

[4] Gupta C, Prakash D (2014). Phytonutrients as therapeutic agents. J. Complement. Integr. Med.

[5] Viegi L, Pieroni A, Guarrera P.M, Vangelisti R (2003). A review of plants used in folk veterinary medicine in Italy as basis for a databank. J. Ethnopharmacol.

[6] Bai N, He K, Roller M, Lai C.S, Shao X, Pan M.H, Ho C.T (2010). Flavonoids and phenolic compounds from *Rosmarinus officinalis*. J. Agric. Food Chem.

[7] Harach T, Aprikian O, Monnard I, Moulin J, Membrez M (2010). Rosemary (*Rosmarinus officinalis* L.). leaf extract limits weight gain and liver steatosis in mice fed a high-fat diet. Planta Med.

[8] Gutierrrez R, Alvarado J.L, Presno M, Perez-Veyna O, Serrano C.J, Yahuaca P (2010). Oxidative stress modulation by *Rosmarinus officinalis* in CCl4-induced liver cirrhosis. Phytother. Res.

[9] Labban L, Mustafa U.E, Ibrahim Y.M (2014). The effects of rosemary (*Rosmarinus officinalis*) leaves powder on glucose level lipid profile and lipid peroxidation. Int. J. Clin. Med.

[10] Al-Nahdi H.S (2012). Effect of *Rosmarinus officinalis* extract on some cardiac enzymes of streptozotocin-induced diabetic rats. J. Health Sci.

[11] Runtuwene J, Cheng K, Asakawa A, Amitani H, Amitani M, Morinaga A, Takimoto Y, Kairupan B.H.R, Inui A (2016). Rosmarinic acid ameliorates hyperglycemia and insulin sensitivity in diabetic rats, potentially by modulating the expression of PEPCK and GLUT4. Drug Des. Dev. Ther.

[12] Lee S.J, Umano K, Shibamoto T, Lee K.G (2005). Identification of volatile components in basil (*Ocimum basilicum* L.). and thyme leaves (*Thymus vulgaris* L.) and their antioxidant properties. Food Chem.

[13] Ezeani C, Ezenyi I, Okoye T, Okoli C (2017). *Ocimum basilicum* extract exhibits antidiabetic effects via inhibition of hepatic glucose mobilization and carbohydrate metabolizing enzymes. J. Intercult. Ethnopharmacol.

[14] Somasundaram G, Manimekalai K, Salwe K.J, Pandiamunian J (2012). Evaluation of the antidiabetic effect of *Ocimum sanctum* in Type 2 diabetic patients. Pharm. Sci.

[15] Suanarunsawat T, Ayutthaya W.D.N, Thirawarapan S, Poungshompoo S (2014). Anti-oxidative, anti-hyperglycemic and lipid-lowering effects of aqueous extracts of *Ocimum sanctum* L. leaves in diabetic rats (2014). Food Nutr. Sci.

[16] Association of Officiating Analytical Chemists (2005). Official Method of Analysis.

[17] Association of American Feed Control Officials (2000). http://www.aafco.org?.

[18] Sandie A (2001). Energy. Small Animal Nutrition.

[19] Emam M.A (2012). Comparative evaluation of antidiabetic activity of *Rosmarinus officinalis* L. and *Chamomile recutita* in streptozotocin-induced diabetic rats. Agric. Biol. J. North Am.

[20] El-Beshbishy H.A, Bahashwan S.A (2012). Hypoglycemic effect of basil (*Ocimum basilicum*) aqueous extract is mediated through inhibition of α-glucosidase and α-amylase activities: An *in vitro* study. Toxicol. Ind. Health.

[21] Hannan J.M.A, Ojo O.O, Ali L, Rokeya B, Khaleque J, Akhter M, Flatt P.R, Abdel-Wahab Y.H.A (2014). Actions underlying antidiabetic effects of *Ocimum sanctum* leaf extracts in animal models of Type 1 and Type 2 diabetes. Eur. J. Med. Plants.

[22] Yan Y.X, Xiao H.B, Wang S.S, Zhao J, He Y, Wang W, Donng J (2016). Investigation of the relationship between chronic stress and insulin resistance in a Chinese population. J. Epidemiol.

